# How variant discovery redefines genetic prevalence: the case of cystine stone disease

**DOI:** 10.1038/s41431-026-02085-y

**Published:** 2026-04-09

**Authors:** Chen-Han Wilfred Wu, Joshua Chang, Katreya Lovrenert, Donald Bodner, Friedhelm Hildebrandt, Fredrick R. Schumacher

**Affiliations:** 1 Department of Urology, Department of Genetics, Hugh Kaul Precision Medicine Institute Heersink School of Medicine, University of Alabama at Birmingham, Birmingham, AL USA; 2 Department of Urology, Department of Genetics and Genome Sciences, Case Western Reserve University School of Medicine and University Hospitals, Cleveland, OH USA; 3 Department of Urology, Case Western Reserve University School of Medicine and University Hospitals, Cleveland, OH USA; 4 Division of Nephrology, Department of Pediatrics, Boston Children’s Hospital, Harvard Medical School, Boston, MA USA; 5 Department of Population and Quantitative Health Sciences, Case Western Reserve University School of Medicine, Cleveland, OH USA

**Keywords:** Rare variants, Genomics, Renal calculi

## Abstract

Cystine stones are caused by pathogenic variants in *SLC3A1* or *SLC7A9*. Our prior study revealed a large gap between genetic and clinical prevalence. With increasing discovery of novel variants, we aim to assess how these impact genetic prevalence estimates. Due to the disease rarity, direct patient recruitment and observation is impractical. We applied a population genetics approach to estimate genetic burden and prevalence. Pathogenic variants were identified from the 2022 Human Gene Mutation Database and intersected with population variants from the 1000 Genomes Project Phase 3. Allele frequency, carrier rate, and affected rate were calculated. Results were compared to prior data, and simulations were performed across varying initial allele frequencies. We identified 116 and 76 novel pathogenic variants in *SLC3A1* and *SLC7A9*, respectively. Pathogenic allele frequencies increased by +0.12% (*SLC3A1*) and 0.16% (*SLC7A9*), leading to fold-changes in genetic prevalence of 1.51x and 2.78x. The combined updated prevalence is 1 in 17,612, a 1.74x increase. Simulations confirmed the fold-change magnitude. In rare diseases, even modest discovery of new variants can significantly increase genetic prevalence. As shown in cystine stone, this helps narrow—but not close—the gap with clinical prevalence. Further efforts are needed to bridge this gap and guide treatment development.

## Introduction

Cystine stone disease is a monogenic disorder resulting from cystinuria, a hereditary metabolic defect characterized by the excessive excretion of cystine in the urine [1]. Cystinuria arises from a defective transport system of the dibasic amino acids in the renal tubules causing cystine reabsorption failure [2]. Coupled with the insolubility of cystine in lower urine pH [3], cystinuria promotes stone formation and is responsible for 1-2% of all kidney stones and 10% of all kidney stones in children [4]. Cystinuria represents the most common cause of monogenic kidney stone disease [5]. Patients suffer from recurrent stone formation and renal insufficiency even with treatment [4, 6, 7].

Cystinuria has a global clinical prevalence of 1 in 7,000 [8]. The genetic etiology of cystinuria is associated with two causal genes [8]. The two genes are *SLC3A1*, located on chromosome 2p21, encoding the heavy subunit rBAT of a renal b(0,+) transporter, and *SLC7A9*, located on chromosome 19q12, encoding its interacting light subunit b(0, + )AT [8–10]. The classification system divides cystinuria into three subtypes based on genetic etiology: type A, caused by pathogenic *SLC3A1* variants; type B, by pathogenic *SLC7A9* variants, and putative type AB, by pathogenic *SLC3A1* and *SLC7A9* variants [11].

Epidemiological studies of cystine stones are limited [12]. Due to its rarity, a cross-sectional or direct observational study is not cost-effective. A 2016 study utilized population genetics approaches to investigate the genetic prevalence of cystine stones revealing a significantly lower calculated genetic prevalence compared to clinical prevalence [13]. In a monogenic disease, such as cystine stone, the genetic and clinical prevalence would be expected to closely align, with minimal or no discrepancy.

As novel variant discovery is an ongoing process, this study aims to evaluate the impact of newly identified pathogenic variants on the genetic prevalence of cystine stone disease, by utilizing an updated database incorporating new pathogenic variants accumulated over recent years.

To quantify this change, we systematically compared current estimates with previous findings. Additionally, simulation analyses were conducted to model the expected impact of variant discovery over time and to compare these projections with real-world data. Through this approach, we seek to gain new insights into the pace and effect of variant discovery on genetic prevalence estimates, and ultimately inform future research strategies for patients with cystine stone.

**Fig. 1:**
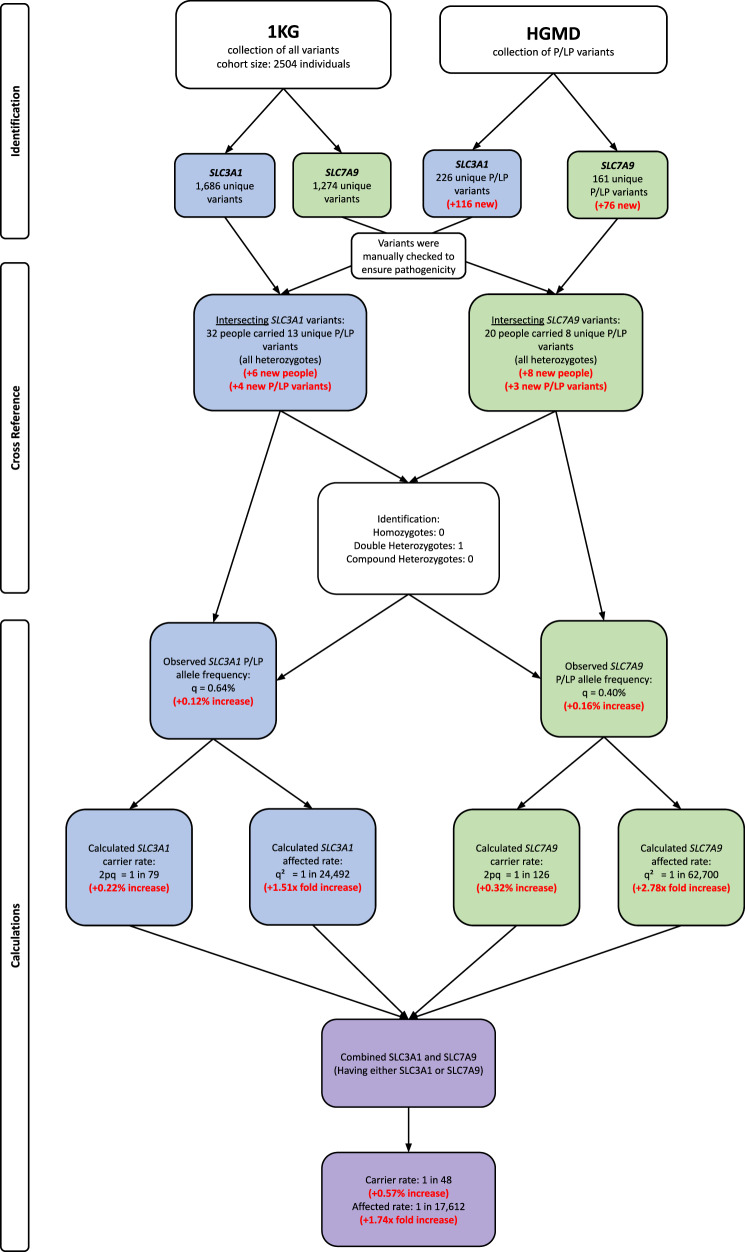
Flowchart to study the 1KG and HGMD database and calculate P/LP allele frequencies, carrier rates, and affected rates of *SLC3A1* and *SLC7A9* for cystine stone. 1KG: 1000 Genomes Project Phase 3. HGMD Human Gene Mutation Database. P/LP Pathogenic/Likely Pathogenic.

**Fig. 2:**
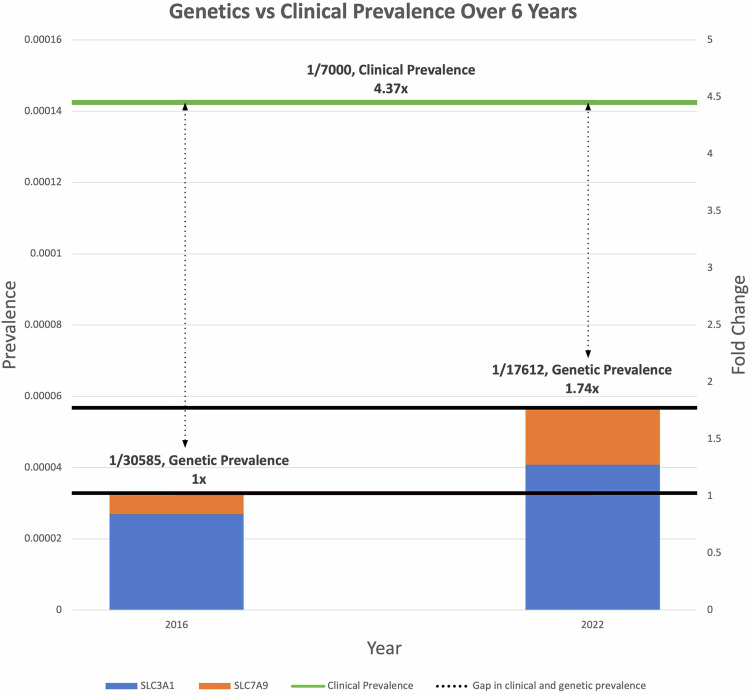
Comparison of change in genetics prevalence of cystine stone over a 6-year period with gap to clinical prevalence. The change in genetics prevalence over 6 years is compared with the corresponding clinical prevalence of cystine stone. X axis represents the years genetic prevalence was estimated, the left y axis represents prevalence of cystine stone, and the right y axis represents fold change, with the genetic prevalence in 2016 serving as 1X baseline. The bar graph shows the prevalence of the causal genes, *SLC3A1* (blue bars) and *SLC7A9* (orange bars) in 2016 and 2022. The genetics prevalence is depicted by summing the genetic prevalence from *SLC3A1* and *SLC7A9*, with prevalence being 1 in 30,585 in 2016 (1x fold change) and 1 in 17,612 in 2022 (1.74x fold change). The clinical prevalence is illustrated by a green line with the prevalence being 1 in 7000 (4.37X of the 2016 genetic prevalence). Over 6 years, the genetic prevalence has increased to 1 in 12,052 (1.74X fold change). However, genetic prevalence remains lower than clinical prevalence with the dotted lines representing the gaps in knowledge regarding cystine stone.

The discovery of new pathogenic variants resulted in an increased pathogenic allele frequency for cystine stones. We found *SLC3A1* pathogenic variants show a 0.12% increase, while *SLC7A9* variants show a 0.16% increase. Despite small increases in allele frequency, the predicted number of affected patients nearly doubled, with a 1.74x increase when combining *SLC3A1* and *SLC7A9* variants (Figs. 1, 2). However, clinical prevalence of cystine stone remains higher than genetic prevalence (Fig. 2). This discrepancy suggests that current genetic knowledge does not fully explain the etiology of cystine stones.

**Fig. 3:**
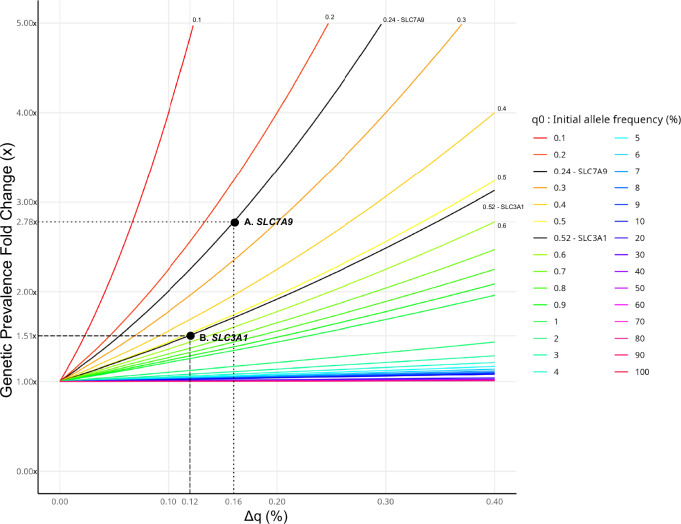
Plot of genetic prevalence fold change as a result of allele frequency change. The fold change of the genetic prevalence was simulated for a range of allele frequency changes. Δq, or change in allele frequency, is the x-axis calculated by the equation Δq=q-q0, where q0 is the initial allele frequency and q is the new allele frequency. x, or genetic prevalence fold change, is the y-axis calculated by the equation x = q^2^/q0^2^, where q^2^ is the new genetic prevalence, and q0^2^ is the initial genetic prevalence. Δq ranges from 0% to 0.4% are illustrated. x as a function of Δq is shown as a set of various colored lines for different simulated values of q0. x for *SLC7A9* and *SLC3A1* are shown as black lines: q0 = 0.24% for *SLC7A9* and q0 = 0.52% for *SLC3A1*. **A** Plot point A represents an observed 2.78x fold change of genetic prevalence for *SLC7A9* with a 0.16% increase of pathogenic allele frequency over 6 years. **B** Plot point B represents an observed 1.51x fold change of genetic prevalence for *SLC3A1* with a 0.12% increase of pathogenic allele frequency over 6 years.

We also demonstrate that, for rare diseases, minor increases in the pathogenic allele frequency can lead to major increases in affected patients (Fig. 3). This highlights major implications for rare diseases as small findings of pathogenic variants can be impactful.

## Methods

Given the rarity of cystine stone disease, direct patient recruitment and observation are impractical. Therefore, we employed a population genetics approach to estimate the genetic burden and disease prevalence [13–18] (Fig. 1).

### Materials

*SLC3A1* and *SLC7A9* variants were identified through the 1000 Genomes Project Phase 3 (1KG) [19] and Human Gene Mutation Database (HGMD) version 2022.4 (Fig. 1).

While gnomAD is a frequently used reference for estimating allele frequencies for a single variant, its composition and lack of individual-level genome data limit its utility for analyses multiple variants on the same gene, or involving two or more genes simultaneously as such in our study regarding cystine stones. Because gnomAD aggregates data from many disease-enriched sequencing studies and can not provide information on which individuals are affected or unaffected, allele frequencies may be distorted by ascertainment bias [20–22]. In fact, gnomAD has officially recommended not to use gnomAD as a general control population due to the lack of information about case numbers for any specific disease [20].

In contrast, 1KG is a comprehensive database of human genetic variants compiled from individuals considered healthy, and obtained through whole-genome sequencing, deep exome sequencing, and dense microarray genotyping [19]. Unlike other aggregate databases, such as GEL and gnomAD, 1KG provides genomic information at the individual level, enabling genetic analyses involving multiple variants on the same gene, and two or more genes simultaneously. For example, gnomAD includes homozygotes for known pathogenic variants in both genes, but without individual-level data it is impossible to determine compound heterozygosity, other cis-trans configuration, or double-gene involvement. The level of individual data in 1KG allows for a more accurate calculation of allele frequencies and carrier rates for these causal genes, such as those implicated in cystine stone formation.

Variant Cell Format (VCF) files aligned to the human reference genome from the 1KG were procured from https://www.internationalgenome.org/data-portal/. The VCFs were narrowed down to the genomic coordinates of *SLC3A1* and *SLC7A9*. Analysis was performed using the High Performance Computing Resource in the Core Facility for Advanced Research Computing at Case Western Reserve University.

The HGMD is a systematic, human-curated collection of identified pathogenic and likely pathogenic variants, derived from continuous screening of peer-reviewed biomedical literature [23]. We identified SNV and CNV variants including small deletions, small insertions, and small indels and obtained a comprehensive list of known pathogenic/likely pathogenic (P/LP) [DM and DM? as stated in HGMD] variants of SLC3A1 and SLC7A9 and acquired from http://www.hgmd.cf.ac.uk/ac/index.php. This DM + DM? method is an all-inclusive approach to maximize estimation of genetic prevalence. We aim to avoid underestimating genetic prevalence by excluding potentially relevant variants. Gross insertions, gross deletions, and complex rearrangements, which may span multiple exons, were not included as they were not possible to detect in 1KG [24]. Here, we use HGMD version 2022.4 and compare it with the version used in our previous publication [13] to assess the impact of newly identified pathogenic variants on genetic prevalence. Since 2016, HGMD has undergone multiple updates, incorporating a substantial number of new variants.

### Allele frequency, carrier rate, affected rate (Genetic prevalence)

Given the rarity of cystine stones and the low genetic prevalence of each pathogenic variant, recruiting a sufficient number of patients and identifying affected individuals is not a practical study design. Therefore, this study adopts a population genetics approach [13–18] as a surrogate to investigate the genetic burden and estimated disease prevalence in the general population. We leveraged Hardy-Weinberg Equilibrium principles [25] to calculate allele frequencies and estimate the affected rate based on the observed carrier rates (Fig. 1).

The *SLC3A1* and *SLC7A9* variants were procured from both databases and intersected to identify P/LP homozygotes, compound heterozygotes, multiple variants in cis or trans, double homozygotes, and double heterozygotes (Fig. 1). Variants found from the intersection were manually reviewed to check the pathogenicity. Benign variants were excluded. The pathogenic allele frequency was calculated for the 1KG [13–17]. Carrier rate and affected rate were estimated based on the Hardy-Weinberg equilibrium of p^2^  +  2pq  +  q^2^  =  1, where q is the frequency of pathogenic variants [25]. Comparative analysis was conducted to juxtapose the calculated allele frequency, affected rate, and carrier rate of *SLC3A1* and *SLC7A9* pathogenic variants for cystine stones with the 2016 publication and provided calculations of genetic prevalence and fold-changes (Table 1, Fig. 2).

**Table 1:** Comparison of *SLC3A1* and *SLC7A9* from 2016 and 2022.

												Genetics vs Clinical Prevalence				
	Observed								Calculated/Expected							
	Carrier Frequency (%)			Allele Frequency (%) [q]					Carrier Rate (2pq)			Affected Rate (q²)**				
	2016	2022	Δ	2016	2022	2022 Standard Error	2022 95% Confidence Interval	Δq	2016	2022	Δ	2016	2022	2022 Standard Error	2022 95% Confidence Interval	Fold change
*SLC3A1* (type A)	26/2504 (1.03%)	32/2504 (1.28%)	+0.25%	26/5008 (0.52%)	32/5008 (0.64%)	0.113%	0.42% - 0.85%	+0.12%	1 in 96	1 in 79	+0.22%	1 in 37,100	1 in 24,492	0.00144%	1 in 14,486 - 1 in 79,198	1.51x
*SLC7A9* (type B)	12/2504 (0.47%)	20/2504 (0.80%)	+0.32%	12/5008 (0.24%)	20/5008 (0.40%)	0.0891%	0.023% - 0.057%	+0.16%	1 in 209	1 in 126	+0.32%	1 in 174,167	1 in 62,700	0.00071%	1 in 33,444 - 1 in 500,746	2.78x
Having either *SLC3A1* or *SLC7A9*	38/2504 (1.51%)	51/2504 (2.04%)*	+0.53%	n/a*					1 in 66	1 in 48	+0.57%	1 in 30,585	1 in 17,612	0.00161%	1 in 11,332 - 1 in 39,505	1.74x
												Clinical prevalence: 1 in 7000				

n/a*: *SLC3A1* and *SLC7A9* are on two diferent chromosomes. The "q" cannot be calculated directly, and each gene should be considered separately.

*: One individual is double heterozygous, causing the redunction of two when adding the numbers of two genes together

The intensity of the shade of red correlates with the increase in percentage.

**Affected Rate (q²) is genetics prevalence

### Statistical analysis for SE and 95% CI

To quantify sampling uncertainty for our estimates, the standard error and 95% confidence interval (CI) was calculated for the allele frequency for SLC3A1 and SLC7A9 using binomial principles [26]. For the affected rate, which is derived from q2, we calculate the standard error and 95% confidence interval (CI) for the affected rate using the delta method [27].

### Simulation of genetic prevalence longitudinal change

To provide a generalized model of changes in genetic prevalence over time, we conducted simulations across a range of initial allele frequencies to evaluate the impact of newly identified pathogenic variants (Fig. 3). The fold-change in genetic prevalence or number of affected individuals (Y-axis) as a function of percent change in allele frequency (X-axis) was modeled by specifying a range of initial allele frequencies (q₀), with different color lines representing each initial q₀ (Fig. 3).

The final allele frequency (q) varied as a function of the initial q₀ and the percent change along the X-axis (Fig. 3). Fold-change in genetic prevalence is defined by FC=q^2^/q_0_^2^ (Fig. 3). q_0_ varied from 0.1% to 100% in three sets; 0.1 to 0.9% in steps of 0.1%, 1 to 10% in steps of 1%, and 20 to 100% in steps of 10%. q, varied from 0% to 0.4% in steps of 0.001%. Fold-change was plotted as a function of Δq=q-q_0_ using R v4.3.0 [28] and ggplot2 v3.4.3 [29] (Fig. 3) (Supplementary Fig. 1).

## Results

### Identification of pathogenic/likely pathogenic (P/LP) genetic variants

The updated HGMD database identified 116 new P/LP *SLC3A1* and 76 new P/LP *SLC3A1* variants for a total of 226 unique P/LP *SLC3A1* variants and 161 unique P/LP *SLC7A9* variants including missense variants, nonsense variants, insertions, deletions, and substitutions, an 105% and 89.4% increase from 2016 (Supplementary Table 1, Fig. 1).

### Identification of genetic variants in the general population

The 1KG database identified 2,504 unrelated healthy individuals with 1,686 unique *SLC3A1* variants and 1,274 unique *SLC7A9* variants [13] (Supplementary Table 2). Variants were classified as single nucleotide variants (SNVs), deletions, or insertions (Supplementary Table 2).

Although most variants are benign, 32 individuals carry 13 unique *SLC3A1* P/LP variants, and 20 individuals carry 8 unique P/LP *SLC7A9* variants based on the updated HGMD (Supplementary Table 3, Fig. 1). Compared to 2016 [13], 7 new *SLC3A1* and *SLC7A9* P/LP variants were added (Supplementary Table 3).

All involved individuals have a heterozygous genotype with no homozygotes or compound heterozygotes. One individual has a double heterozygous genotype in *SLC3A1* and *SLC7A9* (Supplementary Table 3, Fig. 1).

### Allele frequency, carrier rate, affected rate (Genetic prevalence) calculation

Calculations with the new HGMD data show disease-causing alleles to have an allele frequency of 0.64% (+0.12% compared to 2016), carrier rate of 1 in 79, and affected rate of 1 in 24,492 (1.51x of 2016) for *SLC3A1* (Table 1, Fig. 1), and allele frequency of 0.40% (+ 0.16%), carrier rate of 1 in 126, and affected rate of 1 in 62,700 (2.78x) for *SLC7A9* (Table 1, Fig. 1). The combined carrier rate is 1 in 48 and combined affected rate is 1 in 17,612 (1.74x) (Table 1, Fig. 1). Despite increases, there is still a significant gap to the clinical affected rate (genetic prevalence), which is 1 in 7000 (Fig. 2, Table 1).

### Standard error and 95% confidence interval for the estimates

The allele frequency of SLC3A1 (0.64%) had a standard error of 0.113% and 95% CI of 0.42–0.85% while the allele frequency of SLC7A9 (0.40%) had standard error of 0.0891% and 95% CI of 0.023–0.057%. The combined affected rate (1 in 17,612) had a standard error of 0.00161% and a 95% CI of 1 in 11,332 to 1 in 39,505. Even with this wide range of the estimate, the interval of genetic prevalence does not touch the clinical prevalence. In other words, genetic prevalence remains statistically lower than the clinical prevalence (Table 1).

### Longitudinal change simulation and comparison

A model of the increasing P/LP allele frequency over time at various baseline allele frequencies was simulated to analyze the impact on the rate of affected individuals (Fig. 3). The simulation reveals when the initial P/LP allele frequency is low, i.e., rare, a small increase of the allele frequency leads to a significant change in the predicted genetic prevalence (Fig. 3).

This trend is consistent with our analysis of the two cystine stone genes using real-world data, where initial allele frequencies of 0.24% and 0.52% showed that modest increases in allele frequency (+0.16% and +0.12%) resulted in 2.78-fold and 1.51-fold increases in genetic prevalence, respectively (Fig. 3).

## Discussion

We investigated the genetic prevalence of cystine stones using the updated HGMD and compared it with the 2016 study [13]. We demonstrated small increases in observed allele frequencies over 6 years, which resulted in major increases in the estimated affected patients: 1.51x fold-change in *SLC3A1*, 2.78x fold-change in *SLC7A9*, and 1.74x for overall affected rate (Table 1, Fig. 1, Fig. 2).

### New discoveries in P/LP variants lead to fold changes in genetic prevalence

The number of pathogenic variants for *SLC3A1* and *SLC7A9* has increased by 105% and 89.4% (Supplementary Table 1) highlighting the growth of genetic discoveries. Although significant reclassification typically occurs within the first year of initial classification [30], no reclassification of pathogenic variants was observed. This indicates that current pathogenic variants identified have a low likelihood of being downgraded.

Most novel variants are extremely rare (i.e., private) and not seen in the general population. Therefore, allele frequencies of pathogenic variants in the population only show marginal increases with a 0.12% increase in *SLC3A1* and 0.16% increase in *SLC7A9* (Table 1, Fig. 1). Despite small changes, the predicted affected rate nearly doubled, with changes of 1.51x in *SLC3A1*, 2.78x in *SLC7A9*, and 1.74x in overall affected rate (Table 1, Fig. 2, Fig. 3).

The 95% confidence interval range of the combined affected rate (q2) is wide, ranging from 1 in 39,505 to 1 in 11,332 (Table 1). However, even with this broad interval, the upper boundary does not overlap with the current clinical prevalence of cystine stone, 1 in 7000. In other words, the genetic prevalence is still significantly lower than the clinical prevalence even with a sharp 1.74x fold change of the estimated affected rate after adding 116 new P/LP SLC3A1 and 76 new P/LP SLC3A1 variants over a 6 year period.

### Comparing our results with the simulation

The simulated model shows increasing pathogenic allele frequency from an initial low P/LP frequency causes major increases in affected rate, consistent with our real-world data calculations (1 in 30,585 to 1 in 17,612) (Fig. 3, Table 1). As we discover more pathogenic variants, we are able to diagnose more patients and help them manage accordingly, as evidenced by Susswein et al. where they illustrate the usefulness of genetic testing with a high frequency of positive results in 10,000 cases [31]. We uphold the same idea, and further show that for rare diseases, as the knowledge of genetic variants expands, the number of expected patients will exponentially rise (Fig. 3), as shown in the curve of our simulation. However, this exponential trend is only true when the initial pathogenic variants’ frequency is low i.e., rare (Fig. 3, red/yellow lines). When the pathogenic allele frequency is high, the trend of increase is less pronounced (Fig. 3, blue/purple/pink lines). For rare diseases, small improvements in understanding rare genetic diseases can have major implications for future patients.

### Gaps in knowledge

Despite the huge increases, the calculated genetic prevalence of cystine stone (1 in 17,612) remains lower than the global clinical prevalence (1 in 7,000) [8] (Fig. 2, Table 1). This indicates the etiology of cystine stone is still more than what current genetic knowledge can explain (Fig. 2). This finding underscores the complexity of precise genetic causes of cystine stones and highlights the need for further research to elucidate other factors involved in the pathogenesis of cystine stones.

### Potential explanations and future research direction

Various factors may potentially explain the gap (Fig. 2), including undiscovered genes, novel variants, and other modification factors.

### Potential undiscovered genes

Detection rate for *SLC3A1* and *SLC7A9* variants in children with cystinuria was 54% for *SLC3A1* and 25% for *SLC7A9* [32]. No pathogenic variants were detected in *SLC3A1* or *SLC7A9* for at least 5–10% of adult patients [33]. Genes other than *SLC3A1* and *SLC7A9* could be responsible for causing cystine stones [34].

### Potential novel variants

Significant number of variants found in patients with cystine stones remain uncharacterized due to the uncertainty of their role in the disease process [11], suggesting that our lack of awareness of potential variants could explain the disparity [35]. Pathogenic variants may be undetected because they are located deep in intronic regions, potentially affecting splice sites, 5’ promoter regions, and 3’ polyadenylation regions without modifying the peptide sequence [34].

### Structural variants (SVs) and copy number variants (CNVs)

The 1KG and other population databases, such as gnomAD and GEL, are based primarily on short-read sequencing technologies [19]. This reliance on short-read sequencing could contribute to the observed discrepancy between genetic and clinical prevalence. Larger structural variants (SVs) and copy number variants (CNVs) are often not detectable using short-read sequencing methods [36]. Although, the proportion of patients attributed to CNVs for SLC3A1 and SLC7A9 is unknown, by counting each variant once, the unique SVs and CNVs together account for 17% and 11% of pathogenic variants of SLC3A1 and SLC7A9, respectively [35]. Future studies incorporating long-read sequencing technologies to detect larger SVs and CNVs may help to more accurately capture the full spectrum of pathogenic variants and potentially narrow the gap between genetic and clinical prevalence estimates.

### Mode of inheritances (MOIs)

Pathogenic SLC3A1 variants are generally associated with an autosomal-recessive (AR) MOI whereas SLC7A9 variants result in broad clinical variability with either AR or autosomal dominant (AD) MOI [37, 38]. In cases with heterozygous carriers of SLC7A9, patients exhibited AD with incomplete penetrance and reported no cystine stones [38, 39]. Although monoallelic carriers have been hypothesized to develop stones, current evidence does not support cystine stone formation in heterozygous individuals [37, 38]. Therefore, while cystinuria may follow either AR or AD inheritance, cystine stone disease itself remains an AR disease. However, not all variants are the same. There is always a possibility that an allele more severe than currently reported could cause haploinsufficiency and present with stone. If these pathogenic alleles were identified and reported, depending on its prevalence in the general population, our estimate of genetic prevalence of cystine stone would be expected to increase.

### Consanguinity

Another consideration is the potential of the consanguinity, either affecting at the genotype or phenotype assessment sides. A study in Saudi Arabia demonstrated the impact of consanguinity in cystine stones reporting parental consanguinity in 78.6% of affected individuals, highlighting the increased risk and the potential for higher disease prevalence within specific populations [40]. While the findings in our study, where no homozygous or compound heterozygotes pathogenic variants were found, suggests that consanguinity is unlikely to materially bias genetic prevalence estimates, its potential impact on clinical prevalence in specific populations remain a consideration when interpreting comparison with clinical estimates.

### Strength and limitations

We leverage population genetic methods to analyze two databases to address the question of cystine stone prevalence, making the difficult and costly task for rare diseases feasible.

Compared to aggregate data, utilizing 1KG allows us to access individual-level data, allowing for identification of homozygotes, compound heterozygotes, multiple variants in cis or trans, double homozygotes, and double heterozygotes—thereby making this study feasible.

Despite these strengths, there are limitations that we would like to address:

Although 1KG is widely regarded as a representative population database, it remains a sampling of the global population. The relatively small sample size of 1KG (2504 individuals) limits the precision of prevalence estimates of cystine stone. This sampling will introduce sampling biases and errors, as evidenced with our calculations for standard error and 95% CI for allele frequency and affected rate (Table 1). At the current stage of genetics, it is not yet feasible to sequence every individual; thus, reliance on sampling is unavoidable. If a larger database providing individual-level data from unaffected populations were available, it would offer a more representative sampling rate. In addition, a reasonable future direction will be to stratify these comparisons by ancestral populations once reliable estimates of clinical cystine stone prevalence by race or ethnicity become available.

Additionally, 1KG data is based primarily on short-read sequencing, which inherently only detects SNV and short indels, and has limited ability to detect SVs and larger CNVs, including exon-level or gene-level deletions and duplications. Due to this limitation, this study had to exclude any known SVs and larger CNVs from HGMD. Future studies incorporating long-read sequencing data can meaningfully contribute to our understanding of the full spectrum of pathogenic variants as well as narrow the gap between genetic and clinical prevalence of cystine stone.

The HGMD professional version accessed was proprietary and not accessible publicly, though HGMD provides other accessible versions. This study does not include novel pathogenic variants identified after 2022. As the HGMD is continuously updated, repeated studies are required to evaluate novel variants and their effects.

## Conclusions

Our study aims to assess how novel P/LP genetic variants impact genetic prevalence estimates. We re-quantify the genetic prevalence of cystine stone based on the latest genetic data, and compare previous calculations to reveal the longitudinal trend.

Over 6 years, genetic discoveries of novel variants resulted in a small increase in pathogenic allele frequency in the general population (+0.12% for *SLC3A1* and +0.16% for *SLC7A9)*. The increases led the predicted number of affected patients to nearly double (1.51x for SLC3A1, 2.78x for SLC7A9, and 1.74x for overall affected rate, with a 95% CI from 1 in 11,332 to 1 in 39,505) (Fig. 1, Fig. 2). Small increases in allele frequency can lead to major increases in the number of affected individuals, highlighting the opportunity that increased genetic knowledge can have major implications for patients with rare diseases.

The substantial increase in identified pathogenic variants over the past six years has led to a major rise in the estimated genetic prevalence of cystine stone disease, from 1 in 30,585 to 1 in 17,612 (Table 1, Fig. 2). This progress suggests that the gap between clinical and genetic prevalence is narrowing. However, the updated genetic prevalence (1 in 17,612, even with a wide 95% confidence interval from 1 in 11,332 to 1 in 39,505) still remains significantly lower than the clinical prevalence (1 in 7000), underscoring persistent gaps in our understanding of the full etiology of cystine stone disease (Fig. 2). Although advancements have been made, these findings highlight that we are not yet fully capturing the complexity of its etiology, and considerable work remains. Continued efforts to investigate both genetic and non-genetic contributors will be essential to inform future diagnostic, treatment, and prevention strategies.

## Supplementary information

Supplementary Table 1

Supplementary Table 2

Supplementary Table 3

Supplementary Figure 1

Supplementary Figure 2

## Data Availability

Data required for analyses are available publicly. Access to the 1000 Genomes Project Phase 3 (1KG) and Human Gene Mutation Database (HGMD) database can be found below. 1000 Genomes Project Phase 3 (1KG): https://www.internationalgenome.org/data-portal/sample. Human Gene Mutation Database (HGMD): https://www.hgmd.cf.ac.uk/ac/index.php
