# Supplementary material for: How variant discovery redefines genetic prevalence: the case of cystine stone disease
Source: Eur J Hum Genet. 2026 Apr 9;34(7):956–63. doi: 10.1038/s41431-026-02085-y (PMC13341753; doi:10.1038/s41431-026-02085-y)
Supplement: Supplementary file 1 — Supplementary Table 1 [file 41431_2026_2085_MOESM1_ESM.pdf]

Supplementary Table 1. Comparison of variant classification of *SLC3A1* and *SLC7A9* unique variants in HGMD database

					SLC3A1 (type A)			*SLC7A9* (type B)		
**Unique Pathogenic Variants in HGMD**					**2016**	**2022**	**% change**	**2016**	**2022**	**% change**
**SNV**		**Exon**	**Coding**	**Missense**	81 (73.6%)	136 (60.2%)	55 (+67.9%)	49 (51.7%)	83 (51.6%)	34 (+88.6%)
				**Nonsense**	10 (9%)	30 (13.3%)	20 (+200%)	4 (2.3%)	9 (5.6%)	5 (+350%)
				**Synonymous**	0	0	0	0	2 (1.2%)	2 (+∞)
			**UTR**		0	0	0	0	0	0
		**Intron**			0	18 (8.0%)	18 (+∞)	0	16 (9.9%)	16 (+∞)
**CNV**	**Deletion**	**Exon**	**Coding**	**Inframe**	1 (0.9%)	3 (1.3%)	2 (+200%)	3 (3.5%)	5 (3.1%)	2 (+66.7%)
				**Frameshift**	11 (10%)	18 (8%)	7 (+63.6%)	20 (23.5%)	29 (18%)	9 (+45%)
			**UTR**		0	0	0	0	0	0
		**Intron**			0	1 (0.44%)	1 (+∞)	2 (2.3%)	3 (1.9%)	1 (+50%)
	**Insertion**	**Exon**	**Coding**	**Inframe**	0	0	0	2 (2.3%)	2	0
				**Frameshift**	5 (4.5%)	15 (6.6%)	10 (+200%)	4 (4.7%)	9 (5.6%)	5 (+125%)
			**UTR**		0	0	0	0	0	0
		**Intron**			0	1 (0.44%)	1 (+∞)	1 (1.1%)	1 (0.62%)	0
	**Complex Substitutions**				2 (1.8%)	4 (1.8%)	2 (+100%)	0	2 (1.2%)	2 (+∞)
**Total number of Pathogenic Variants**					110 (100%)	226 (100%)	116 (+105%)	85 (100%)	161 (100%)	76 (+89.4%)

HGMD: Human Gene Mutation Database; SNV: Single Nucleotide Variant;
UTR: Untranslated Region;

The intensity of the shade of red correlates with the increase in percentage.
