# Supplementary material for: How variant discovery redefines genetic prevalence: the case of cystine stone disease
Source: Eur J Hum Genet. 2026 Apr 9;34(7):956–63. doi: 10.1038/s41431-026-02085-y (PMC13341753; doi:10.1038/s41431-026-02085-y)
Supplement: Supplementary file 2 — Supplementary Table 2 [file 41431_2026_2085_MOESM2_ESM.pdf]

Supplementary Table 2. Classification of *SLC3A1* and *SLC7A9* unique variants in the 1KG database

<table>
<tr><th colspan="4">Unique Variants in 1KG</th><th>SLC3A1</th><th>SLC7A9</th></tr>
<tr><td rowspan="5">SNV</td><td rowspan="4">Exon</td><td rowspan="3">Coding</td><td>Missense</td><td>102 (6%)</td><td>69 (5.4%)</td></tr>
<tr><td>Nonsense</td><td>0</td><td>0</td></tr>
<tr><td>Synonymous</td><td>7 (0.4%)</td><td>7 (0.5%)</td></tr>
<tr><td colspan="2">UTR</td><td>47 (2.7%)</td><td>11 (0.8%)</td></tr>
<tr><td colspan="3">Intron</td><td>1429 (84.7%)</td><td>1124 (88.2%)</td></tr>
<tr><td rowspan="4">Deletion</td><td rowspan="3">Exon</td><td rowspan="2">Coding</td><td>Inframe</td><td>0</td><td>0</td></tr>
<tr><td>Frameshift</td><td>0</td><td>0</td></tr>
<tr><td colspan="2">UTR</td><td>0</td><td>0</td></tr>
<tr><td colspan="3">Intron</td><td>63 (3.7%)</td><td>40 (3.1%)</td></tr>
<tr><td rowspan="4">Insertion</td><td rowspan="3">Exon</td><td rowspan="2">Coding</td><td>Inframe</td><td>0</td><td>0</td></tr>
<tr><td>Frameshift</td><td>0</td><td>0</td></tr>
<tr><td colspan="2">UTR</td><td>0</td><td>0</td></tr>
<tr><td colspan="3">Intron</td><td>38 (2.2%)</td><td>23 (1.8%)</td></tr>
<tr><td colspan="4">Complex Substitutions</td><td>0</td><td>0</td></tr>
<tr><td colspan="4">Total number of Variants</td><td>1686 (100%)</td><td>1274 (100%)</td></tr>
</table>

1KG: 1000 Genomes Project Phase 3; SNV: Single Nucleotide Variant;
UTR: Untranslated Region
