# Supplementary material for: How variant discovery redefines genetic prevalence: the case of cystine stone disease
Source: Eur J Hum Genet. 2026 Apr 9;34(7):956–63. doi: 10.1038/s41431-026-02085-y (PMC13341753; doi:10.1038/s41431-026-02085-y)
Supplement: Supplementary file 3 — Supplementary Table 3 [file 41431_2026_2085_MOESM3_ESM.pdf]

Supplementary Table 3. Characteristics of intersected genetic variants (pathogenic variants) of *SLC3A1* and *SLC7A9* in 1KG and HGMD

										(a) 2016			(b) 2022		
	Chr	Position	Variant Identifier	Ref Allele	Var Allele	C. nomenclature	P. nomenclature	Exon number	Type	In HGMD?	# of people carrying this variant (all heterozygotes)	Observed Allele Frequency (%)	In HGMD?	# of people carrying this variant (all heterozygotes)	Observed Allele Frequency (%)
	chr2	44502931	rs199700629	G	A	c.257G>A	p.Arg86Gln	1/10	missense	N	-	-	Y	2	2/5008 (0.04%)
	chr2	44507965	rs142482973	C	T	c.541C>T	p.Arg181Trp	2/10	missense	N	-	-	Y	2	2/5008 (0.04%)
	chr2	44507990	rs140317484	C	T	c.566C>T	p.Thr189Met	2/10	missense	Y	6	6/5008 (0.12%)	Y	6	6/5008 (0.12%)
	chr2	44508585	rs542026439	C	T	c.670C>T	p.Gln224*	3/10	nonsense	N	-	-	Y	1	1/5008 (0.02%)
	chr2	44513202	rs141587158	T	C	c.797T>C	p.Phe266Ser	4/10	missense	Y	7	7/5008 (0.14%)	Y	7	7/5008 (0.14%)
	chr2	44528215	rs121912697	G	A	c.1085G>A	p.Arg362His	6/10	missense	Y	2	2/5008 (0.04%)	Y	2	2/5008 (0.04%)
	chr2	44528224	rs567478582	G	A		c.1094G>A	6/10	missense	Y	1	1/5008 (0.02%)	Y	1	1/5008 (0.02%)
SLC3A1	chr2	44531449	rs184648701	T	G	c.1304T>G	p.Met435Arg	7/10	missense	N	-	-	Y	1	1/5008 (0.02%)
	chr2	44539726	rs187962930	T	C	c.1334T>C	p.Ile445Thr	8/10	missense	Y	2	2/5008 (0.04%)	Y	2	2/5008 (0.04%)
	chr2	44539746	rs201502095	C	T	c.1354C>T	p.Arg452Trp	8/10	missense	Y	1	1/5008 (0.02%)	Y	1	1/5008 (0.02%)
	chr2	44539758	rs139251285	C	T	c.1366C>T	p.Arg456Cys	8/10	missense	Y	1	1/5008 (0.02%)	Y	1	1/5008 (0.02%)
	chr2	44539773	rs144162964	T	C	c.1381T>C	p.Tyr461His	8/10	missense	Y	4	4/5008 (0.08%)	Y	4 (^)	4/5008 (0.08%)
	chr2	44539792	rs121912691	T	C	c.1400T>C	p.Met467Thr	8/10	missense	Y	2	2/5008 (0.04%)	Y	2	2/5008 (0.04%)
	SLC3A1 total # of Unique Variants									9			14		
	SLC3A1 Carriers Summary										26	26/5008		32	32/5008
	chr19	33321545	rs146815072	G	A	c.1445C>T	p.Pro482Leu	13/13	missense	Y	1	1/5008 (0.02%)	Y	1	1/5008 (0.02%)
	chr19	33334874	rs45628833	C	T	c.978-17G>A		9/12*	splicing	N	-	-	Y	6 (^)	6/5008 (0.12%)
	chr19	33350791	rs147344717	C	T	c.829G>A	p.Val277Met	8/13	missense	N	-	-	Y	1	1/5008 (0.02%)
	chr19	33353409	rs531029519	C	T	c.562G>A	p.Val188Met	5/13	missense	Y	1	1/5008 (0.02%)	Y	1	1/5008 (0.02%)
SLC7A9	chr19	33353427	rs79389353	C	T	c.544G>A	p.Ala182Thr	5/13	missense	Y	5	5/5008 (0.1%)	Y	5	5/5008 (0.1%)
	chr19	33355112	rs79987078	G	A	c.368C>T	p.Thr123Met	4/13	missense	Y	2	2/5008 (0.04%)	Y	2	2/5008 (0.04%)
	chr19	33355167	rs121908480	C	T	c.313G>A	p.Gly105Arg	4/13	missense	Y	3	3/5008 (0.06%)	Y	3	3/5008 (0.06%)
	chr19	33359415	rs200753692	G	A	c.26G>A	p.Arg9Gln	2/13	missense	N	-	-	Y	1	1/5008 (0.02%)
	SLC7A9 total # of Unique Variants									5			8		
	SLC7A9 Carriers Summary										12	12/5008		20	20/5008

* Intron number

One individual has a double heterozygote with *SLC7A9* c.978-17G>A with two pathogenic variants in *SLC3A1* c.1381T>C, denoted by (^).

Red shaded regions indicate new variants compared to previous publication (PMID 37561200).

Unshaded regions indicate old variants found in the previous publication
