# Supplementary material for: How variant discovery redefines genetic prevalence: the case of cystine stone disease
Source: Eur J Hum Genet. 2026 Apr 9;34(7):956–63. doi: 10.1038/s41431-026-02085-y (PMC13341753; doi:10.1038/s41431-026-02085-y)

Supplementary Figure 1A/B. Simulated models of affected rate fold-change by allele frequencies

A. Percent change in allele frequency from 0% to 100%

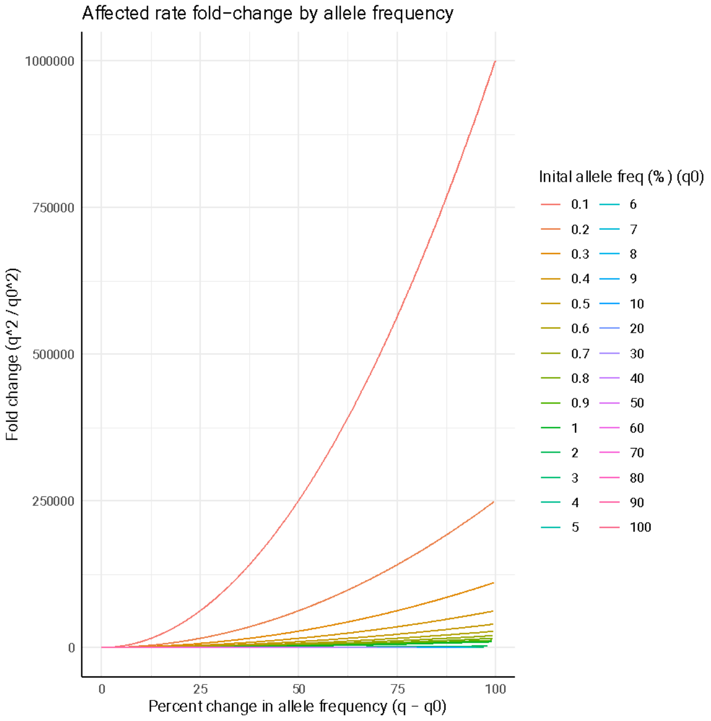

B. Percent change in allele frequency from 0% to 1%

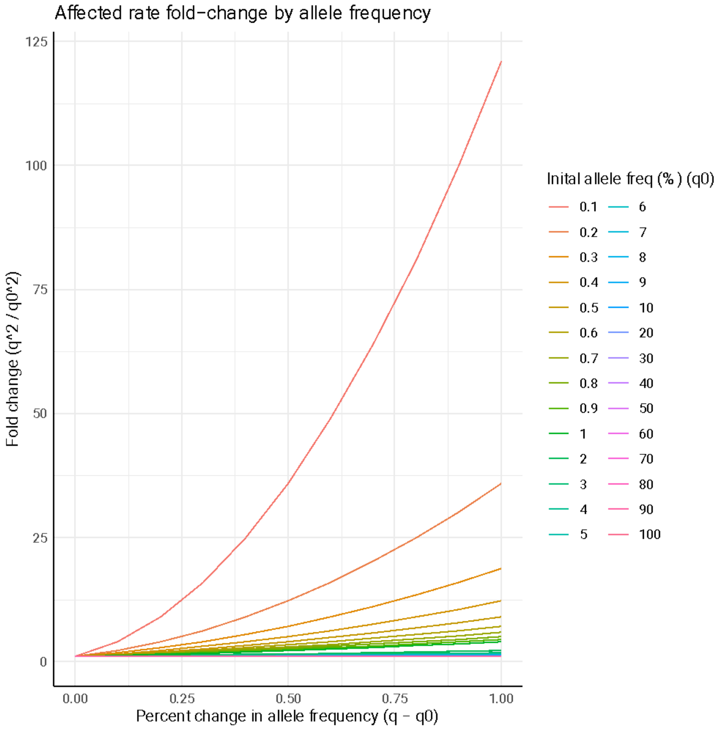

Supplement: Supplementary file 4 — Supplementary Figure 1 [file 41431_2026_2085_MOESM4_ESM.pdf]
